# Sirtuins Modulation: A Promising Strategy for HIV-Associated Neurocognitive Impairments

**DOI:** 10.3390/ijms23020643

**Published:** 2022-01-07

**Authors:** Izchel Figarola-Centurión, Martha Escoto-Delgadillo, Gracia Viviana González-Enríquez, Juan Ernesto Gutiérrez-Sevilla, Eduardo Vázquez-Valls, Blanca Miriam Torres-Mendoza

**Affiliations:** 1 Doctorado en Genética Humana, Departamento de Biología Molecular y Genómica, Universidad de Guadalajara, Guadalajara 44340, Mexico; figarola.centurion.izchel@gmail.com; 2 Laboratorio de Inmunodeficiencias y Retrovirus Humanos, Centro de Investigación Biomédica de Occidente, Instituto Mexicano del Seguro Social, Guadalajara 44340, Mexico; martha.escotod@gmail.com (M.E.-D.); juan251995@hotmail.com (J.E.G.-S.); 3 Centro Universitario de Ciencias Biológicas y Agropecuarias, Universidad de Guadalajara, Guadalajara 44600, Mexico; 4 Departamento de Disciplinas Filosófico, Metodológicas e Instrumentales, Centro Universitario de Ciencias de la Salud, Universidad de Guadalajara, Guadalajara 44340, Mexico; gvivigoen@gmail.com; 5 Microbiología Médica, Centro Universitario de Ciencias de la Salud, Universidad de Guadalajara, Guadalajara 44340, Mexico; 6 Generación de Recursos Profesionales, Investigación y Desarrollo, Secretaria de Salud, Jalisco, Guadalajara 44100, Mexico; eduardo.vazquezvalls@jalisco.gob.mx

**Keywords:** sirtuin, SIRT1, SIRT3, SIRT2, HIV, resveratrol, ER-stress, mitochondrial dysfunction, neurodegeneration, HAND

## Abstract

HIV-Associated neurocognitive disorder (HAND) is one of the major concerns since it persists in 40% of this population. Nowadays, HAND neuropathogenesis is considered to be caused by the infected cells that cross the brain–blood barrier and produce viral proteins that can be secreted and internalized into neurons leading to disruption of cellular processes. The evidence points to viral proteins such as Tat as the causal agent for neuronal alteration and thus HAND. The hallmarks in Tat-induced neurodegeneration are endoplasmic reticulum stress and mitochondrial dysfunction. Sirtuins (SIRTs) are NAD+-dependent deacetylases involved in mitochondria biogenesis, unfolded protein response, and intrinsic apoptosis pathway. Tat interaction with these deacetylases causes inhibition of SIRT1 and SIRT3. Studies revealed that SIRTs activation promotes neuroprotection in neurodegenerative diseases such Alzheimer’s and Parkinson’s disease. Therefore, this review focuses on Tat-induced neurotoxicity mechanisms that involve SIRTs as key regulators and their modulation as a therapeutic strategy for tackling HAND and thereby improving the quality of life of people living with HIV.

## 1. Introduction

In 2020, the human immunodeficiency virus (HIV) infected more than 37 million people around the world, and 73% of this population was under antiretroviral therapy [1]. This combined antiretroviral treatment (cART) has expanded their life span. However, comorbidities have emerged, provoking changes in the central nervous system (CNS). Neuroimaging techniques have provided evidence of morphological changes in the brain of people living with HIV (PLWH). Additionally, reports showed the presence of HIV in cerebrospinal fluid and post-mortem brain tissues even when the viral load was undetectable in blood [2].

Collectively, these alterations are denominated as HIV-associated neurocognitive disorders (HAND), and are categorized depending on severity according to the Frascati criteria as asymptomatic neurocognitive impairment (ANI), mild neurocognitive disorder (MND), or HIV-associated dementia (HAD) [3]. The global prevalence of HAND was estimated at around 40%, with the milder form being the most common among the HAND patients [4,5]. Frascati criteria have been extensively utilized for identifying each level of HAND; they apply neuropsychological tests and exclude any comorbidity condition that could cause cognitive impairments. ANI and MND are diagnosed when the performance of a minimum of two cognitive domains is at least 1 SD below the mean, evaluating no fewer than five cognitive domains, which can include attention, language, executive and motor function, working memory, speed of information processing, and learning. Nevertheless, in ANI, the daily activities are not affected, while MND patients show mild impairments in everyday activities. Finally, people diagnosed with HAD have a performance of 2 SD below the mean in two cognitive domains and evident difficulty in the achievement of day-to-day activities [3,6]. HAND causes a spectrum of cognitive impairment, motor dysfunction, and behavioral and emotional disorders [7,8,9,10,11,12,13,14].

**Figure 1:**
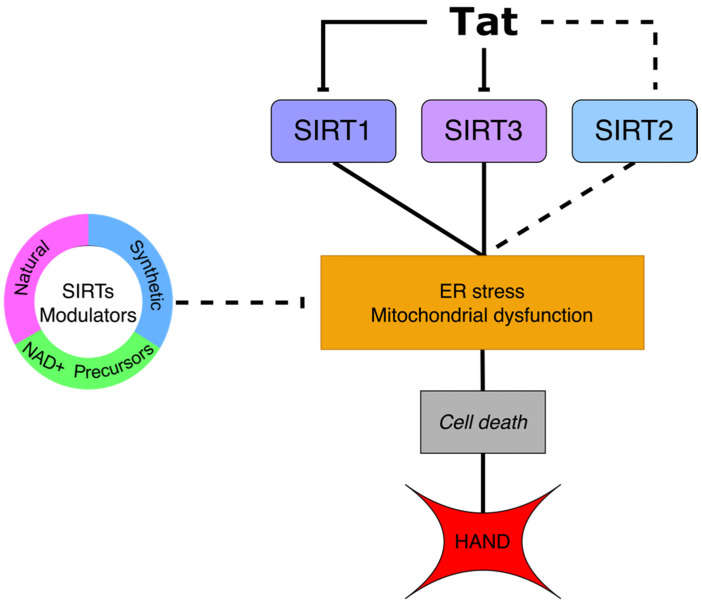
Tat-mediated SIRT1 and SIRT3 inhibition lead to endoplasmic reticulum (ER) stress and mitochondrial dysfunction. These cellular events induce activation of the intrinsic pathway that causes cell death, a hallmark in HAND. In the case of SIRT2, the effect of Tat interaction remains unknown (dotted line). SIRTs modulators have been used in other neurodegenerative disorders. Therefore, they could contribute to Tat-induced neurotoxicity mitigation.

Being at risk for developing HAND depends on the CD4+ count, time of infection, plasma viral load, and history of AIDS-defining illness [15]. Nowadays, HAND is considered to be caused by either the neurotoxicity of cART or the viral proteins secreted by infected cells or a combination of both [2]. HIV cannot infect the neuron, but it can cross the BBB through infected monocytes that produce viral proteins that provoke neurotoxicity [16]. In animal models and cell lines, it has been observed that transactivator of transcription (Tat) induces alterations in CNS [17,18,19,20,21]. A hallmark in Tat-induced neurodegeneration is endoplasmic reticulum (ER) stress [22,23] and mitochondrial dysfunction, which are characterized by disruptions in mitophagy [18], mtDNA [20], fusion and fission [24], and energy metabolism [25,26]. SIRTs, NAD+-dependent deacetylases, are involved in molecular mechanisms of these mitochondrial processes [27,28]. Furthermore, the data have revealed a cross interaction between Tat and SIRTs, causing their inactivation [29,30,31]. In this review, we focus on the approaches that the viral protein Tat employs to induce neurotoxicity through disruption of SIRTs-regulated pathways. Conversely, we propose the use of SIRTs modulators such as NAD+ precursors, and natural and synthetic compounds, as a possible therapeutic strategy, together with ART, to improve cognitive deficits in PLWH; hence, currently, there is not an effective treatment to tackle this illness (Figure 1).

## 2. HAND and the Long-Term Exposure to ART

The prevalence of HAND, even in the era of ART, has directed attention towards antiretrovirals with higher CNS-penetration-effectiveness (CPE), expecting this classification would have tackled the problem [32]. Nonetheless, treatments with this characteristic proved to be associated with HAND, especially efavirenz [33,34,35]. Furthermore, other antiretrovirals with different CPE have been reported as neurotoxic agents [36,37,38]. The mechanisms that govern ART-induced neurotoxicity depends on the class of drug. Initially, the nucleoside analog reverse-transcriptase inhibitors (NRTIs) were found to cause mitochondrial aberrations by the depletion of mtDNA due to the analogy of the active site of the HIV reverse transcriptase and the DNA polymerase gamma (PolG) that causes inhibition of mtDNA replication [39], which can be related to disruption of mitochondrial biogenesis. Additionally, the exposure of protease inhibitors such as lopinavir showed an increase in oxidative stress mediated by high ROS levels in neural cells and activation of endogenous antioxidant response [38]. In the case of ritonavir, it was found that it induces endoplasmic reticulum (ER) stress and mitochondrial outer-membrane permeabilization (MOMP) [40]. Finally, the data suggesting ART-mediated cognitive performance improvement is still controversial [38,41,42]; hence, some studies did not find a beneficial association, while Siangphoe et al. [42] concluded in a meta-analysis that ART actually reduces the risk of developing neuronal disorders such as HAND. Nevertheless, the study did not include in the analysis the type of ART of each patient, which raises a question about if any specific antiretroviral or a combination of them is causing any neurocognitive deficit. Additionally, the diagnosis of HAND was not performed by the same scale, resulting in possible variation at the moment of identification of these conditions and the sensibility of the cognitive test to detect ANI [42]. As discussed above, time living with HIV seems to contribute to the development of neurocognitive impairment, which seems to converge with the exposure of ART and its toxicity. Therefore, special consideration must be taken to adjuvant drugs for proper prevention or treatment against HAND, considering not only the side effect of ART but also the concomitant cell alterations induced by viral proteins, such as Tat.

## 3. Transactivator of Transcription Tat

Tat, a small regulatory protein, is encoded in *tat* gene of the HIV genome by two separated exons that after alternative splicing, and the protein can be fully generated. The length depends upon the viral strain, which ranges from 86 to 101 amino acids. It is composed of N-terminal acidic or proline-rich, cysteine-rich, hydrophobic core; basic and glutamine-rich domains; and a RGD motif. Tat has residue variability, causing a range of activation and inhibitory results on host proteins and gene expression. Only the residue W11 and sequence 49RKKRRQRRR57 are well conserved residues due to the essential function of secretion and uptake of Tat bystander cells [43].

### Secretion and Internalization of Tat

Infected cells that penetrate the blood–brain barrier (BBB) can release viral protein as Tat in an unconventional manner because it lacks a signal peptide to transit across the Golgi apparatus or the endoplasmic reticulum [44]. Once Tat is recruited to the cell membrane, its basic domain, and residue W11 interact with phosphatidylinositol-4,5-bisphosphate (PI(4,5)P2) [45]. The subsequent mechanisms have not been completely elucidated. On the one hand, reports have shown that Tat can oligomerize and form pores in the plasma membrane. On the other side, binding to PI(4,5)P2 allows insertion of residue W11 into the cell membrane; then, it is translocated to the extracellular matrix by an unclear process [44,45,46]. Finally, studies have reported exosomes contained Tat [44,47]; nevertheless, exosomal Tat seems to lose its neurotoxicity property [19].

Extracellular Tat enters the cell by endocytosis. After internalization, Tat translocates into the cell cytoplasm. Conformational change takes place as a result of low pH, allowing exposure of W11 residue, which interacts with the endosomal membrane [48]. Additionally, the calcium coming from endolysosome-resident two-pore channels assists Tat regarding the scape of endolysosome [49]. Further, Ruiz et al. found a reduction of 70% in the uptake of Tat by bystander cells mediated by substitution in the basic domain (R57S), proving the pivotal relation of residue R57 in the cellular internalization of Tat [50]. In fact, Tang et al., attributed Tat-induced synaptic damage to its ability to penetrate cell membranes [19]. Indeed, a short sequence of Tat is being used as a cell penetrating peptide for delivering drugs in other illnesses like cancer [51]. It seems that Tat uses different strategies to get inside and out of the cell, which makes it a significant target for prevention of neuronal alterations. Therefore, we detailed the mechanisms involved in the following sections.

## 4. Tat and Mitochondria

Mitochondria are organelles involved in several cellular processes, including energy production in an oxidative phosphorylation-manner, apoptosis signaling, calcium homeostasis [52], and cellular aging regulation [53]. Studies have focused on the role of mitochondria in neuronal disorders not only in Parkinson’s disease (PD) or Alzheimer’s disease (AD) [54] but also in HAND [55]. Sanna et al. [56] demonstrated presence of mitochondrial dysfunction mediated by high HIV RNA load, which was determined by low TCA cycle and oxidative phosphorylation protein levels in brain regions of HIV patients [56]. It was observed that Tat remained near to the mitochondria [57,58], suggesting cellular distribution is related to organelle alterations. Accordingly, other findings revealed that Tat-exposed cells present mtDNA damage [20,59] and alteration in its methylation patterns [60], mitochondrial membrane potential reduction [23] changes in size and morphology of the organelle, fusion-fission triggering [24], increased mitochondrial ROS [61,62], mitophagy disruption [17,57], mitochondrial functions dysregulation [17,61] calcium homeostasis disturbance [61,63,64], and apoptosis activation [23,58,61,65,66].

### 4.1. Tat and Fusion-Fission Dynamics

Mitochondria is a highly dynamic organelle that undergoes fusion and fission to maintain functional processes. In neurons, energetic demand triggers mitochondria fusion/fission-mediated distribution to axons and dendrites. The fusion of the OMM is regulated by GTPases mitofusin 1 and 2 (MFN1, MFN2), while the fusion of the inner mitochondrial membrane (IMM) is controlled by optic atrophy protein 1 (OPA1). Meanwhile, mitochondrial divisions events are mediated by dynamin-related protein 1 (DRP1), a GTPase from the dynamin superfamily. It has been observed in Drp1 and Mnf2 knockout mice that cells were unable to properly distribute mitochondria [67]. 

Tat-Tg mice showed small fragmented mitochondria and in Tat-exposed cortical neurons exhibited an increase in DRP1 and calcineurin, suggesting that Tat induces mitochondrial fragmentation by dysregulation of Ca^2+^ homeostasis, which triggers calcineurin-mediated DRP1 activation [24].

### 4.2. Mitophagy Disruption

Mitochondrial is the powerhouse of the cell, and therefore their maintenance is crucial for cellular physiology. Damaged mitochondria trigger quality control pathways to restore mitochondria network and energy metabolism. When the dysfunctional mitochondria persist, cells undergo mitophagy, which involves degradation of aberrant organelles [68]. The phosphatase and tensin homolog (PTEN)-induced putative kinase 1 (PINK1) is a crucial regulator of mitophagy. During basal conditions, PINK1 is translocated into the inner mitochondrial membrane and degraded. Nevertheless, under loss of mitochondrial membrane potential, PINK1 is accumulated in OMM, where it initiates mitophagosome formation. To label non-functional mitochondria, PINK1 autophosphorylates and recruits parkin to the mitochondria surface. Then, ubiquitination of proteins in OMM takes place by parkin E3 ligase activity, followed by mobilization of autophagy receptors such as sequestome 1 (SQSTM1) in order to enclose damaged mitochondria into the mitophagosome [68]. 

In mouse microglia, Tat increases the expression of PINK, indicating activation of mitophagy. In addition, data showed not only higher levels of mitophagy sensor proteins but their active translocation to mitochondria in Tat-exposed human primary neurons [57]. In both research groups, SQSTM1 was found to increase, suggesting mitophagy flux impairment, which was also confirmed by the accumulation of mitophagosomes observed by fluorescence reporter system [57]. It is suggested that incomplete mitophagy might occur because fusion of lysosomes to the mitophagosomes is blocked due to immature phagosome formed or cells cannot balance degradation them at the same rate they are formed [17]. It seems that further investigations are needed to clarify the cause of the obstruction of clearance of damaged mitochondria.

The blockage of mitochondria quality control leads to production of proinflammatory cytokines, contributing to neuroinflammation. However, gene silencing of pink1 causes prevention of HIV-Tat-induced mitophagy and thus accumulation of mitophagosomes, but it is unsuccessful to restore ATP production rate [17]. In this regard, searching for the key regulator of mitophagy proteins that could be dysregulated is necessary since manipulation of mitophagy markers did not result in restoration of mitochondria functions, which in turn provoked neurodegeneration.

### 4.3. Tat-Induced Apoptosis

The reported data shows that Tat-exposed cells undergo extrinsic [65] and intrinsic apoptosis pathways [23]. In the first one, the signaling process starts in the cell membrane and is triggered by stimulation of death receptors, followed by activation of caspases to initiate cell death. The intrinsic apoptosis pathway is also referred to as the mitochondrial apoptotic pathway because MOMP is required to trigger caspase signaling that induces apoptosis [54]. Recently, most studies have acknowledged the significant role of the mitochondrial pathway in Tat-induced apoptosis and its crosstalk to ER stress and unfolded protein response (UPR) [23,69,70].

The cell, in the presence of apoptotic stimuli, translocates BCL-2-associated X protein (BAX) from cytoplasm to the OMM, where it oligomerizes and interacts with BCL-2 antagonist/killer (BAK) acting as pores after binding with pro-apoptotic activators [71]. The BH3-only proteins such as BCL-2- interacting mediator of cell death (BIM), BH3-interacting domain death agonist (BID), and p53-upregulated modulator of apoptosis (PUMA) are pro-apoptotic members of the B cell lymphoma 2 (Blc-2) family that interact with BAK and BAX leading to the release of intermembrane space proteins such as cytochrome c and thus MOMP [71]. Consequently, these proteins initiate caspases activation leading to apoptosis. Note that MOMP is not only involved in the initiation of the apoptotic pathway, it is also related to the mtDNA release and proinflammatory signaling. The survival mechanism is regulated by the anti-apoptotic proteins from the same Bcl-2 family such as BCL-2 and B cell lymphoma extra-large (BCL-XL). These molecules bind to pro-apoptotic proteins, avoiding their interaction with BAX/BAK and the pore formation [71]. Hence, dysregulation of the BCL-2 proteins is an indication of the intrinsic apoptotic pathway as well as mitochondrial dysfunction. In Tat-exposed erythroleukemic cell line (K562) [58], human retinal microvascular endothelial cells [69], astroglioma cell [66], and human neuroblastoma cell [18], the mRNA levels of BCL-2 were lower compared to controls. Che et al. also found that the proteins levels of BAX, BAK, and cytochrome c were increased by Tat in human retinal pigment epithelial cells (ARPE-19) [69]. According to these findings, Tat acts as a pro-apoptotic molecule, increasing MOMP by causing macropores formation in the OMM, which is also confirmed in reports of disruption of the mitochondrial membrane potential [23,58,70]. 

Interestingly, Campestrini et al. [23] found that mRNA of Bcl-2 levels were upregulated after 72 h Tat-treated human T lymphocyte cell line (Jurkat). Nevertheless, they used HIV-Tat clade C, which has a change R57S compared to clade B and that interferes with the cell uptake of Tat [50]. In addition, several reports suggested differences in cell effects between clade B and C, with the last being less neurotoxic [72]. Therefore, partial mitochondrial damage is suggested, or an incomplete MOMP where the undamaged mitochondria show high levels of BCL-2 [54], but further studies are required to confirm this hypothesis. Conversely, ER stress is observed in the intrinsic apoptotic pathway, which derives into a UPR [23]. The ER is largely known for its functions of protein traffic, folding, and maturation. ER stress is acquired when a stimulus causes an alteration in functional protein process, leading to unfolded or misfolded proteins. In order to counter malfunction, ER triggers the UPR where signaling cascades are activated to reconfigure upstream protein production, protein translocation to ER, and autophagy, to restore proteostasis or induce apoptosis if ER homeostasis is not reached. The pathways involved in UPR are conducted by the membrane ER proteins PERK, IRE1a, and ATF6a. PERK phosphorylates eukaryotic translation initiation factor 2 subunit-α (eIF2α) so protein synthesis and unfolded protein influx can be reduced. eIF2α induces translation of ATF4 mRNA, which stimulates gene expression of proteins required for reduction-oxidation balance; autophagy; and apoptosis such as CCAAT/enhancer-binding protein homologous protein (CHOP), growth arrest, and DNA damage-inducible protein (GADD34). CHOP regulates GADD34, which at the same time modulates death receptor 5 (DR5), which recruits caspase 8 for cleavage-mediated activation of BID, one of the pro-apoptotic proteins in the mitochondrial pathway discussed above [73]. Another signaling pathway is the one regulated by the IRE1 dissociation from GRP78 and its RNase activity activation. Then, IRE1 targets the mRNA of the X-box-binding protein 1 (XBP1), removing a 26-nucleotide intron that allows for the expression of the activated form of XBP1. This protein is an upregulator of other genes related to folding, secretion, and elimination of misfolded proteins. In addition, IRE1 can undergo a process called regulated IRE1-dependent decay (RIDD), where mRNAs are degraded, including the DR5, an anti-apoptotic mechanism. However, conditions of prolonged stress reduce RIDD effectiveness, causing cell death. Lastly, the ATF6 cascade begins with its transport to the Golgi apparatus and cleavage to release a fragment denominated ATFp50, which translocates to the nucleus and induces gene expression in a similar way that XBP1 but with the difference that ATFp50 also modulates Golgi apparatus biogenesis [73]. In this context, it has been reported that Tat-induced ER stress leads to unfolded protein response (UPR) activation identified by the gene expression increase of IRE1, PERK, and ATF6, and at the same time, signatures were observed of mitochondrial dysfunction mediated by the loss of mitochondrial membrane potential and caspase 12 and caspase 3 activation [23]. Additionally, in human brain, microvascular endothelial cells Tat-exposure effects include increased protein levels of IRE1, PERK, ATF6, and Bip/GRP78, which resulted in ROS increase [70]. 

Cells suffer from Ca^2+^ influx increase through Tat interaction with *N*-methyl-d-aspartate receptor (NMDAR) [63]. Nevertheless, strategies to block this receptor have failed to mitigate HAND [74], indicating that Tat uses other pathways to disrupt Ca^2+^ homeostasis. Ca^2+^ is stored in ER in normal conditions, and its release is modulated by different stimuli. Recently, evidence has indicated that both ER and mitochondria interconnect through a complex known as a mitochondria-associated ER-membranes, where cellular processes occur, including Ca^2+^ transport and induction to cell death during long-term cellular stress [75]. Ca^2+^ signaling is the most recognized interaction between these two organelles, with the assistance of IP3 receptors [76]. The ER-mitochondria communication seems to figure as a feedback loop. Increased cytosolic Ca^2^+ levels due to ER stress-mediated release provoke disruption in mitochondrial membrane potential, causing an increase in ROS production. ER stress can be induced by impaired protein maturation process triggered by high levels of mtROS. Furthermore, mitochondrial Ca^2+^ uptake induces the opening of permeability transition pore (mPTP) and release of cytochrome c, which drives activation of caspases [75], signifying that Ca^2+^ signaling between ER and mitochondria regulates cellular respiration. Therefore, an increase in cytoplasmatic Ca^2+^ concentration could derivate into cell demise.

Observations in Tat-exposed embryonic rat hippocampal neurons showed an increment in cytosolic Ca^2+^ and mitochondrial uptake, which cause membrane depolarization and cell death [64]. Tat-induced intracellular Ca^2+^+ increase is characterized by an initial transitory stage IP3-mediated and a second prolongated period mediated by NMDARs in fetal human brain neurons [77] and the transient receptor potential canonical channel in rat Nacc neurons [74] that leads to cell death. These findings confirm that Tat impacts mitochondria-ER communication through the dysregulation of Ca^2+^ homeostasis and imbalance of pro and anti-apoptotic proteins, leading to mitochondrial dysfunction, which in turn provokes apoptosis. As mentioned before, clinical trials have been performed to tackle Tat-mediated Ca^2+^ dysregulation using NMDAR antagonists without significant results in mitigating the progression of HAND [74]. The reason behind these results might be the fact that calcium dysregulation is just one of Tat-mediated effects, which is caused by multiple upstream events. Therefore, a strategy could be to tackle an event where most of disrupted pathways converge and are key to neurodegeneration, such as mitochondrial dysfunction, and to tackle its regulator molecules such as sirtuins.

## 5. Sirtuins

Sirtuin1–7 are part of a family of proteins class III histones deacetylases. All SIRTs are NAD+-dependent enzymes and participate in a variety of cellular events such as gene expression, DNA repair, and aging [78]. They present a diversity of functions and cellular distribution because of structural differences in N- and C-terminal. The conserved catalytic core of SIRTs has two subdomains: Rossmann-fold and a zinc-binding domain. Substrates and NAD+ bind to the active cleft between the two subdomains. The interaction between catalytic core, substrate, and NAD+ induces a conformational change that leads to active cleft closing [79]. The well characterized catalytic activity is lysine deacetylation, but several experiments concluded that SIRT5 can also eliminate succinyl and malonyl groups and that SIRT4 is an ADP-ribosyltransferase [80]. 

**Figure 2:**
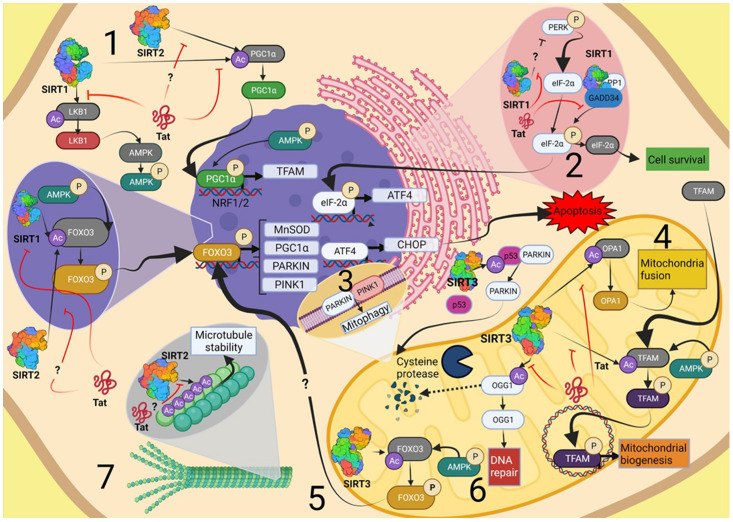
SIRTs-regulated pathways where viral protein Tat induces cytotoxicity. (**1**) Mitochondria biogenesis. SIRT1 participates in this cellular process through several deacetylation reactions. First, SIRT1 deacetylates LKB1, which in turn activates AMPK. This kinase phosphorylates FOXO3, which is also deacetylated by SIRT1 to induce transcription of genes such as PGC1α. SIRT1-mediated deacetylation of PGC1α promotes the expression of genes involved in mitochondria biogenesis, such as Nrf1-2 and TFAM. SIRT2 could participate in this process by interaction with PGC1α in the same way that SIRT1 (**2**) UPR. SIRT1 influences the phosphorylation of eIF-2α in two ways, one of which is through modulating phosphorylation levels of PERK, which activates eIF-2α. The second pathway is when SIRT1 regulates dephosphorylation/deacetylation of eIF-2α via GADD34/PP1 complex. (**3**) Mitophagy. The SIRT1/3-mediated deacetylation of FOXO3 promotes transcription of PINK1, a key protein in mitophagy. SIRT3 deacetylates p53/Parkin complex, allowing parkin translocation to the OMM to initiate mitophagosome formation. (**4**) Mitochondria fusion. SIRT3-mediated deacetylation of OPA1 promotes mitochondrial fusion and modulation of mtDNA copies, morphology, and size of mitochondria. (**5**) Antioxidant response. Expression of MnSOD is induced by SIRT3-mediated deacetylation of FOXO3. (**6**) mtDNA repair. SIRT3-mediated deacetylation prevents degradation of OGG1, which in turn DNA eliminates oxidized guanine 8-oxoG to repair the mtDNA. (**7**) Microtubule stability. SIRT2 interacts with α-tubulin, which is part of the cytoskeleton. Acetylation levels correlate with microtube stability. Image created with BioRender.com (Toronto, ON, Canada).

SIRT1 and SIRT2 are found in the nucleus and cytoplasm, while SIRT3 is mainly located in mitochondria. Indeed, this helps to restrict their substrates since SIRTs showed no preference in protein sequence. However, acetyllysine residue must be within a loop or α-helices to be accessible to deacetylation reaction [80]. Despite the apparent substrate non-specificity of SIRTs, it was revealed that only SIRT1, SIRT2, and SIRT3 can deacetylate Tat [31], which proves a direct interaction that could lead to changes in these SIRTs function towards other substrates. Therefore, in this review, we pay attention to only the above-mentioned three molecules, and the SIRTs-regulated pathways are shown in Figure 2.

### 5.1. Sirtuins in Neurodegenerative Diseases 

Sirtuins 1–7 are expressed in the brain at different degrees. SIRT1 and SIRT2 are the most frequent in this tissue, but SIRT1 is highly expressed in neurons, while SIRT2 is abundant in oligodendrocytes. The sirtuins 3–5 is the second group to be found in the brain, and the least common are the SIRT6-7. SIRT1 is predominantly located at the nucleus, and SIRT2 is distributed in the cytoplasm, while SIRT3 is by preference abundant in mitochondria but with some isoforms being in cytoplasm and nucleus [81]. SIRTs expression differs depending upon brain areas, age, and pathological conditions and has been related to neurodegenerative disorders such as PD and AD. PD pathogenesis is characterized by aggregation of α-synuclein, which can be reduced in mice brain PD model due to SIRT1 overexpression [82]. However, inhibition of SIRT2 mitigates α-synuclein toxicity in human neuroglioma cells [83]. On the other hand, SIRT1, SIRT3, and SIRT6 expression is reduced in the brain of patients with AD [84]. Moreover, SIRT1 [85] and SIRT3 [86] protein concentrations are reduced in the cerebral cortex of individuals with AD. Both SIRT1 and SIRT3 dysregulation has been related to cognition performance [85,87].

### 5.2. SIRT1

SIRT1 participates in several cellular processes, including cell metabolism, DNA repair, mitochondrial biogenesis, apoptosis [88,89], circadian timing system [90], gene silencing through the interaction with DNA methyltransferase 1 [91], heterochromatin formation [92], cell cycle progression [93], and oxidative stress response [94]. Due to the interactions with numerous molecules, SIRT1 has drawn attention for its role in many diseases, including cancer [95], type 2 diabetes [96], and neurodegenerative disorders [97,98].

#### 5.2.1. SIRT1 in ER Stress and UPR

Recently, findings have revealed that SIRT1 plays an intrinsic role in ER stress mitochondria apoptotic pathway regulation [27,99,100]. In primary chondrocytes, pharmacological and genetic inhibition of SIRT1 leads to phosphorylated PERK increase and downstream proteins such as eIF-2α and CHOP [27]. Similarly, in cardiac cells, depletion of SIRT1 increases protein levels of phosphorylated eIF-2α, ATF4, GADD34, and CHOP [99]. However, overexpression or activation of this sirtuin causes abrogation of cell death in both studies. Additionally, an increase of acetylated forms of both PERK [27] and eIF-2α [99] was observed. Nevertheless, Ghosh et al. [100] reported that even without ER stress stimulus, SIRT1 depletion is involved in PERK UPR branch activation by regulating levels of phosphorylated eIF-2α; in addition, they found evidence of physical interaction between SIRT1, CHOP, and GADD34 [100] that was later confirmed by evidence showing formation of an arsenite-induced Sirt1/GADD34/PP1/eIF-2α complex and nuclear SIRT1 translocation to cytoplasm, which leads to GADD34-mediated dephosphorylation/deacetylation of eIF-2α and dephosphorylation of SIRT1 [101]. Collectively, this information indicates that SIRT1 can act as a regulator of apoptosis; hence, the complex GADD34/PP1 is a repressor of eIF-2α, leading to restoration of ER function when cellular stress has been solved [73]. Moreover, it has been suggested that the role of SIRT1 in cell proliferation is mediated by its location [102], which is also dependent on the cell type [103]. However, it is needed to elude the mechanisms that cell types use against different types of stressors and the compensatory responses that are activated. 

#### 5.2.2. SIRT1 and Mitochondrial Dysfunction

Mitochondria dysfunction emerges from the disruption in their metabolic and energetic functions, as well as dysregulation in their quality control events, including organelle fission-fusion, mitochondrial biogenesis, and mitophagy. The dynamics between fusion and fission are essential for cell adaptation and organelle distribution upon several conditions, such as mitophagy and mitochondria biogenesis [104]. SIRT1 regulates mitochondrial biogenesis through several deacetylation reactions. The serine/threonine-protein kinase STK11(LKB1) is activated by SIRT1, leading to the phosphorylation of AMP-activated protein kinase (AMPK). Subsequently, FOXO3 is phosphorylated and deacetylated by SIRT1 to promote transcription of PGC1α, which induces transcription of nuclear transcription factors (Nrf1-2) and the transcription factor A (TFAM), which is related to mtDNA replication and transcription [28]. Paradoxically, SIRT1 has been related to mitophagy, but the detailed mechanisms are still being determined. Some studies have revealed that activators such as resveratrol (RV) and SRT1720 induce mitophagy by transcriptional induction of PINK1 through FOXO3 SIRT1-mediated acetylation [105,106]. Additionally, SIRT1 is required for mitochondrial fragmentation by acting upon the cytoskeleton in the context of Ca^2+^ dysregulation [107]. These findings underpin the fact that SIRT1 is a key modulator in mitochondrial dysfunction.

#### 5.2.3. SIRT1 and Tat

In HIV infection, SIRT1 is required for Tat Lys50 deacetylation since Tat-trans-acting responsive element (TAR) complex formation only takes place in its unacetylated form, but Tat acetylation is required to proceed with the transcription. Once the process is completed, the same Tat molecules can be recycled through SIRT1-deacetylation to initiate another TAR complex [31,108]. Interestingly, the SIRT1-Tat interaction is independent of Tat acetylation state [31], which suggests Tat-induced SIRT1 inhibition takes place when Tat recycling is no longer need it [108]. However, there are some questions about the inhibition mechanisms because it is unclear if allosteric mechanisms are taking place and if there is a concentration dependence of the inhibition. However, it is notable that Tat-induced SIRT1 inhibition leads to hyperactivation of T cells [30]. As a central player of several cellular processes, Tat-mediated SIRT1 inhibition might be involved in cell dysregulation that in turn causes neuronal loss. Therefore, the activation of this sirtuin should be considered to tackle HAND.

### 5.3. SIRT2

Although SIRT2 concentrations are higher in the cytosol, during cell cycle G2/M transition they are translocated to the nucleus, where they regulate H4K20 methylation by deacetylation of histone H4K16, which is key in chromosome compaction [109]. Paradoxically, there is evidence of cell proliferation disruption and cell cycle arrest in overexpressing SIRT2 lung cancer cells [110]. In the cytoplasm, SIRT2 interacts with α-tubulin, which is part of the cytoskeleton. Acetylation levels correlate with microtubule stability, and in SIRT2-overexpressed cerebellar granule cells of slow Wallerian degeneration mice, disruption of hyperacetylation and resistance to axonal degeneration was observed [111]. However, it was found that SIRT2^-/-^ mice cause axonal damage accompanied by low glutathione levels reduced ATP, reduced mtDNA concentration, and resulted in high expression of SIRT1 compared to wild type [112], signifying disruption of mitochondria homeostasis. In line with these results, Liu et al. [113]. observed that in striatum SIRT2^-/-^ mice cells, several mitochondrial proteins have higher acetylation levels than WT, including those involved in energy production. The possibility that SIRT2 acts in the mitochondria was also confirmed in the same group since SIRT2 could be localized in the mitochondria of Wt mice brains and murine embryonic fibroblasts [113]. Furthermore, SIRT2^-/-^ mice cortex revealed that depletion of this sirtuin causes mitochondrial size change, increased levels of acetylated PGC1α, and the downregulation of expression of mitochondrial fusion-related related genes Mfn1, Mnf2, and Opa1 [113]. Conversely, under stress conditions, HepG2 SIRT2-Wt cells decreased DRP1; meanwhile, SIRT2-catalytic inactivated cells did not [114]. In the context of oxidative stress, SIRT2 overexpression increases cell viability and induces FOXO3a-mediated SOD2 expression in neuroblastomas cells [115]. On the other hand, SIRT2^-/-^ MFs showed an increment in ROS and mitophagy key regulator proteins, PINK1, and parkin, as well as LC3B, a maker for impairment of damaged mitochondria clearance [113]. The neuroprotective effects discrepancies of SIRT2, which might be the consequence of the several compensatory responses the cell takes under different stimuli. Nonetheless, the role of SIRT2 in mitochondrial homeostasis is notable. The fact that Tat physically interacts with this sirtuin and that a marker of Tat-induced neurotoxicity is mitochondrial dysfunction can lead us to hypothesize a possible modulation in the same manner of SIRT1. Therefore, future investigations are necessary to decipherer the relationship between SIRT2 and Tat.

### 5.4. SIRT3

The energy metabolism pathways carried out in the mitochondria, such as the tricarboxylic acid pathway and ATP generation, are influenced by SIRT3 [116,117]. Therefore, SIRT3 is related to aging, cancer, cardiac dysfunction, and neurodegenerative diseases [117]. SIRT3 is the main sirtuin in the mitochondria, despite evidence that SIRT4 and SIRT5 are also present in this organelle. In addition, SIRT3 acts under oxidative stress, and it can regulate mitochondrial biogenesis, autophagy/mitophagy, and apoptosis in diverse cell types [116].

#### 5.4.1. SIRT3 and Antioxidant Response 

Overexpression of SIRT3 reduces ROS in MEFs [118] and cardiomyocytes by upregulating gene expression of manganese superoxide dismutase (MnSOD) and by deacetylation of FOXO3a and activating glutathione pathway [119,120,121]. Catalytic inactivated SIRT3 or SIRT3^-/-^ cells showed disruption of the GSH:GSSG ratio [119,120]. This alteration could be linked to SIRT3-mediated deacetylation of isocitrate dehydrogenase 2 (IDH2), which is a source of the NADP required in GHS/GSSG reactions [120]. Furthermore, depletion of SIRT3 is accompanied by loss of the mitochondria membrane potential in MEFs [118]. The evidence reveals that SIRT3 is involved in ROS mitigation in the cell through the regulation of gene expression and activation of proteins of the antioxidant response.

#### 5.4.2. SIRT3 and Mitochondrial Biogenesis and mtDNA Integrity

PGC1α is a pivotal player in mitochondrial biogenesis, hence it regulates the expression of NFR1 that, in turn, enhances the transcription of TFAM, thereby promoting the production of mitochondrial proteins [28]. SIRT3 silencing downregulates the gene expression of PGC1α, as well NRF1, TFAM, and SIRT1 [122], which are PGC1α activators [28]. On the other hand, SIRT3 protects mtDNA through the regulation of OGG1 and Lon proteins [123,124]. OGG1 repairs DNA, eliminating oxidized guanine 8-oxoG caused by ROS. SIRT3 deacetylates OGG1 to induce its activity since the silencing of SIRT3 irradiated cells exhibited an increase of 8-oxoG compared to SIRT3 Wt [123]. Indeed, it was observed that SIRT3 deacetylation prevents OGG1 degradation by calpain, a calcium-dependent nonlysosomal cysteine protease [123]. Mitochondria metabolism not only depends on OGG1 repair activity but also on the replicative events of mtDNA. Lon, a mitochondrial protease, is involved in nonfunctional protein degradation and mtDNA preservation and replication, through the regulation of TFAM [125]. There is evidence indicating that Lon is deacetylated by SIRT3 in breast cancer cell line [124]. Accumulating evidence suggests that SIRT3 modulates mitochondria biogenesis in a transcriptional manner and modulates the stability of mitochondrial proteins required for maintenance of mtDNA.

#### 5.4.3. SIRT3 and Mitophagy

As discussed before, mitochondria dynamics and mitophagy are linked through mitochondria fission-fusion events. OPA1, an elemental protein in fusion processes, has been related to mtDNA copy number. It was reported that SIRT3 induces the GTPase activity of OPA1 via its deacetylation [118], promoting mitochondria fusion, modulating their morphology and the genetic amount in the mitochondria network. When mitochondria do not recover from damage, mitophagy signaling is activated [68]. There are reports that SIRT3 upregulated mitophagy key proteins, such as parkin, under stress conditions [126]. Indeed, it was determined that SIRT3 physically interacts with PINK1 and parkin and that its overexpression reduced the amount of acetylation of these proteins [127]. Additionally, it was suggested that the activation of mitophagy by stressors is mediated through deacetylation of FOXO3a [126]. In SIRT3^-/-^ mouse myocardia cells, impairment of Parkin-mediated mitophagy was detected due to the increase of the complex p53/parkin, which avoids parkin mitochondrial translocation [128]. In addition, it has been reported that the N-terminal of SIRT3 interacts with p53 [86]. Furthermore, overexpression of SIRT3 in a PD model increases LC3II and Beclin 1 expression, indicating autophagosomes formation, also confirmed by microscopy. The autophagy SIRT3-mediated activation is through LKB1/AMPK/mTOR [129]. Collectively, these data imply that SIRT3 actives autophagy/mitophagy to rescue cells from apoptosis; hence, SIRT3 overexpression can abrogate apoptosis via deacetylation of mitochondria p53 [86]. 

#### 5.4.4. SIRT3 and Tat

In microglia, Tat downregulates the expression of SIRT3 and protein levels of superoxide dismutase-2 (SOD2), and of CAT accompanied by SIRT3 low enzymatic activity. Of note, Thangaraj et al. [29] found that expression of SIRT4 and SIRT5 was not altered, thus confirming that Tat modulates only SIRT3, causing an oxidative environment in the cells. However, overexpression of SIRT3 inhibited the Tat-induced dysregulation in microglia, including abrogation of expression of proinflammatory cytokines [29]. According to the data, activation of SIRT3 must be included as a possible pathway for future investigations focused on preventing and ameliorating the effects of Tat-induced neurodegeneration.

## 6. SIRT Modulation

### 6.1. Inhibitors

As a result of the role of SIRTs in many cellular processes related to diseases such as cancer and metabolic disorders, a variety of molecules have been studied as SIRTs inhibitors. Sirtinol, suramin, and EX-527 have shown SIRTs inhibition activity [78]. Among the inhibitors for SIRT3 are the thieno [3,2-d] pyrimidine-6-carboxamide derivatives that have been demonstrated to inhibit SIRT1 as well [79]. Most of the inhibitors interact with SIRTs in an active, cleft-binding manner [79], meaning that the catalytic site is no longer available during this interaction. Nevertheless, some molecules act via a different mechanism, such as the case of SirReals or Sirtuin rearranging ligands that are considered allosteric inhibitors of SIRT2 and allow for the binding of NAD+. AGK2 is an inhibitor of SIRT2 with neuroprotective results, from reduced cell death and ROS to decreasing inflammation [103]. Not only small molecules are being studied as SIRTs inhibitors, but also pseudopeptides are the target of peptide- based drug design studies [79]. The suramin, used in the treatment of onchocerciasis and trypanosomiasis, is an inhibitor of SIRT1, SIRT2, and SIRT5 [79]. It has also been investigated as HIV treatment without a beneficial outcome [130]. The use of inhibitors to treat HIV infection was postulated in the early years of research because it was found that Tat requires K50 deacetylation for viral transactivation and that it was SIRT1-dependent. Nonetheless, later works indicated that viral protein Tat reduces deacetylation activity of SIRT1, its gene expression, and protein levels in vitro [131] and induces SIRT1 post-transcriptional silencing mediated by miRNA-34a and miRNA-138 [132]. These findings propose a particular requirement at different stages of infection, and because of the cART, viral transcription is controlled, so inhibition of SIRTs could lead to concomitant neurodegeneration.

### 6.2. Activators

SIRTs activation promotes cardio and neuroprotection in several conditions such as myocardial ischemia [133], atherosclerosis [134], cardiac hypertrophy [135], Huntington disease [98,136], PD [137], and AD [138]. Nowadays, there are two approaches to active SIRTs. One is to use natural or synthetic compounds to increase expression or their deacetylation activity. Another perspective is to boost the availability of NAD+ through its precursors. Here, we briefly describe the mechanisms of these xenobiotics to encounter cell stressors, and they are summarized in Table 1.

#### 6.2.1. Polyphenols

The molecule of RV was shown to be an activator of SIRT1 [79,139,142,167]. It has been reported that RV ameliorates ROS under oxidative stress [142,143] and inflammatory conditions [139]. It was reported that RV also increases the expression of SIRT3; hence, it activates SIRT1, which in turn deacetylates PGC1a. This transcription factor is influenced by RV to bind to SIRT3 promoter in neurons exposed to high concentrations of manganese [142]. The data suggest that RV rescues cell integrity by not only enhancing mitochondrial biogenesis due to SIRT1-mediated deacetylation of PGC1a and SIRT3-mediated deacetylation of TFAM [142] but also by inducing mitophagy, as is confirmed by the increase of proteins such as PINK1 and parkin in cardiac injury [106]. Furthermore, in Pb-exposed rats, RV was shown to improve spatial learning memory accompanied by cell proliferation and reduction of neuronal apoptosis [141]. As mentioned before, Tat can inhibit SIRT1 activity. Interestingly, high concentrations of RV have been proved to alleviate this effect and to attenuate HIV-1 LTR transactivation [140]. This corroborates the need to further investigate the effect of RV or analogs, not only in HIV infection but also in Tat-induced mitochondria dysfunction and ER stress in CNS.

Piceatannol (PC), a polyphenol found in grapes and other plants such as passion fruit or sugar cane, is a RV analog [144] that is also an active SIRT1 [168]. However, PC structure confers a higher capability to scavenge ROS by modulating other pathways as well [143]. The mechanisms of PC mitigation of ROS involve stimulation of antioxidant enzymes such as SOD and CAT activities [144,145]. Moreover, PC improves mitochondrial functions by increasing complex I activity and ATP content [144]. PC has been demonstrated to induce mitochondrial biogenesis through the increase of expression and protein levels of SIRT1 and PGC1α under gamma-irradiation [144]. On the other hand, apoptosis is reduced by PC increase of anti and pro-apoptotic proteins such as Bcl-2 and BAX, respectively [145]. However, PC exhibits great cytoprotection potential against oxidative stressors; it was observed that at a high concentration, PC promotes depolarization of mitochondrial membrane that leads to cell death. These data indicate that PC can act as an antioxidant or anticancer agent in a dose-dependent manner [143].

Polydatin, a polyphenolic compound isolated from *Polygonum cuspidatum*, protects against lipopolysaccharide-induced endothelial barrier disruption [169], enhances mitochondria biogenesis, increases autophagy, decreases ROS, and reduces apoptosis via activation of SIRT3 in cardiomyocytes under hypoxia [146]. Interestingly, in sulfur mustard exposed L02 cells, SIRT3 expression was unchanged compared to control. Instead, SIRT1 levels were downregulated, and polydatin treatment reversed this condition [147]. This information implies that the polydatin is a xenobiotic that can activate both SIRT1 and SIRT3 but is stressor-dependent.

#### 6.2.2. Other Natural Compounds

Honokiol, a natural compound extracted from Magnolia species [170], activates SIRT3 by direct interaction and upregulating its gene expression in a PGC1a-dependent manner [135], which can suggest an interaction with SIRT1 since it is an activator of PGC1a, a coactivator of antioxidant enzymes [117,171]. In fact, reports propose that honokiol also actives SIRT1 [149] and increases its gene and protein levels [148]. Honokiol has been implicated in ROS mitigation; increased cell viability; and decreased apoptosis [148,149] and protein levels of pro-apoptotic makers such as CHOP, p-PERK, GRP78, and CASP3 [148]. Among the activators of both SIRT1 and SIRT3 are dioscin, pyrroloquinoline quinone (PQQ), and melatonin. Dioscin is a saponin found in some plants that can cross the BBB [151] and exhibits neuroprotection by increasing SIRT1 and SIRT3 expression. Dioscin treatment of neurons can ameliorate ROS production, reduce cell death, and reduce neuroinflammation [138,151]. Indeed, it was observed that dioscin promotes autophagy by increasing Beclin-1 and LC3-II [138], to respond to cellular stressors. PQQ is a natural redox cofactor that can be found in plants. It has ROS scavenger potential and promotes mitochondrial biogenesis by upregulating mRNA of NAMPT, an essential enzyme for the production of NAD+. Subsequentially, this upregulation leads to increase in gene expression of SIRT1 and SIRT3 in HepG2 cells [152], hence increasing NAD+ availability. The following effects of PQQ are the increase of gene expression of PGC1α and downstream targets such as TFAM [152]. In microglia, it was proved that PQQ neuroprotective properties have been reported by activation of PINK1/parkin-mediated mitophagy in rotenone-exposed cells [172]. In the case of melatonin, a neurohormone produced by the pineal gland, it has been observed that SIRT3 activity rises without increasing its expression in an atherosclerosis mouse model [134] or in cadmium-exposed HepG2 cells [153]. This activity increase was accompanied by elevation of LC3II/I ratio, parkin expression, and decrease of mitochondrial TOM20 protein levels and acetylated FOXO3a, signifying that melatonin can induce mitophagy to protect against atherosclerosis injury through the activation of SIRT3/FOXO3a pathway [134] and activation of SOD2 [153]. In addition, like all the SIRT3 activators, melatonin was capable of mitigating mitochondrial ROS [134,153] and ameliorating mitochondria membrane potential loss in macrophages treated with to oxidized low- density lipoprotein [134]. Contrary to the atherosclerosis model, Zhai et al. [133] found that melatonin increased both expression and activity of SIRT3 in myocardial ischemia/reperfusion injury. Interestingly, melatonin also increases protein levels of SIRT1 [133,154], and in the same group observed that when SIRT1 was inhibited or silent, SIRT3 expression was affected but not in the other direction [133]. Lipopolysaccharide-induced oxidative stress in microglia causes a decrease in nuclear Nrf2 and its downstream genes, which is reversed by melatonin SIRT1 interaction [154]. Furthermore, it was reported that melatonin reduces ER stress in the cortex of brain under cerebral hypoxia-ischemia through the reduction of activation of PERK and ATF-6 UPR pathways and the increase in SIRT1 levels [155]. The translocation of SIRT1 to the cytoplasm is related to apoptosis, an observation made in CoCl2-exposed microglia and blocked by melatonin treatment [156]. In agreement with this information, melatonin-mediated SIRT1 activation and nuclear translocation are also related to mitochondrial biogenesis via the increase of PGC1α [157]. Accordingly, SIRT1 pathway is intimately related to SIRT3 in amelioration of stressor-induced mitophagy and therefore must be considered to further investigate both regulations in Tat-exposed neural cells.

Another natural xenobiotic is dihydromyricetin (DH), a flavonoid present in plants such as *Ampelopsis grossedentata*, which has been found to confer protection in liver injury and cardiovascular, dermatological, and neurodegenerative diseases [173]. DH increases SIRT3 protein levels via PGC1α and induces mitophagy in murine chondrocytes [158]. SIRT3 can also be modulated by silybin, an extract from *Silybum marianum*. Silybin induces reduction of ROS; apoptosis; and mitochondria dysfunction, and increases energy metabolism in cells exposed to cisplatin, an anticancer drug that causes acute kidney injury [160]. Additionally, SIRT3 modulates ROS by deacetylation of MnSOD, which is increased by treatment with phloretin, a molecule found in apples and pears [174].

Oxidative modulation can be activated by β-lapachone (LP), a quinone compound extracted from *Tabebuia avellanedae,* which has several beneficial effects, from anticancer to neuroprotective properties. For instance, in a murine of Huntington’s disease, LP increases SIRT1 protein and thus deacetylation of PGC1α and decreases mitochondrial superoxide, meaning that LP can promote mitochondrial biogenesis and ROS amelioration [159]. 

Finally, Tanshinone IIA (TAN), a compound extracted from *Salvia miltiorrhiza,* has been reported to have neuroprotective properties. TAN can activate the LKB1-AMPK pathway by increasing SRT1 activity in mouse bone-marrow-derived mast cells [164]. Moreover, TAN can restore mitochondrial function by preventing mPTP opening and thus intrinsic apoptotic pathway activation in microvascular endothelial cells exposed to a stressor [165]. In line with these data, TAN can reduce Tat-induced cytotoxicity in TZM-bl cells via Nampt activation, which in turn increases NAD+ availability, which subsequently activates SIRT1. This activation cascade is responsible for ROS decrease and inhibition of HIV transactivation [166]. Remarkably, this study provides evidence supporting the role of SIRTs activation in the mitigation of Tat effects on cells.

#### 6.2.3. Synthetic Drugs

Due to the lack of specificity of some natural xenobiotics, synthetic drugs have been developed to increase SIRTs activity, including SRT2104 [175] and SRT1720 [163]. SRT2014 can penetrate the BBB effectively, and it has been involved in neuroprotection by upregulating autophagy [162,175]. SRT1720 has been reported to upregulate key proteins of mitochondrial biogenesis PGC1α and bioenergetic-related protein such as ATP synthetase c [163]. Metformin, a drug used in type 2 diabetes, can increase SIRT3 expression in peripheral blood leukocytes of type 2 diabetes patients and low mitochondria ROS [161].

#### 6.2.4. NAD+ as Co-Substrate of SIRTs

There is great interest in exogenous SIRTs activator compounds; however, other endogenous modulator molecules can be used as SIRTs activators such as nicotinamide riboside (NR) and nicotinamide mononucleotide (NMN), which are precursors of nicotinamide adenine dinucleotide (NAD+). This coenzyme is essential for SIRTs deacetylation activity. Interestingly, even though SIRT1 mRNA levels increase with age in animal brains, there is deterioration in its enzymatic activity, which is related to the NAD+ decline [84]. Notably, not all SIRTs are NAD-dependent in the same manner since SIRT1 and SIRT3 have higher Km values than the other SIRTs, meaning that their function is favorable when NAD+ levels are higher [176]; therefore, increasing NAD+ concentration may restore their enzymatic activity. NAD+ syntheses from NMN or from NR are different because the first requires a one-step reaction while the other involves a phosphorylation step to be transformed into NMN and then NAD+, mediated by NMN/NAMN adenylyltransferase (NMNAT). These reactions occur in the cytoplasm, but recent evidence has shown the mitochondrial uptake of NR and NMN, which it is supported by the localization of NMNAT in this organelle as well [176]. 

NMN has been reported to increase NAD+ concentration [137] and improve cognition by inhibiting the JNK pathway, synapse loss, and neuroinflammation in a murine model of AD [177]. Additionally, in presence of a mitochondrial complex I inhibitor, NMN rescues mitochondrial ATP production and prevents apoptosis [137]. Conversely, NR supplementation also can increase NAD+ levels in brain NR and has been evaluated in AD models, resulting in attenuation of cognitive impairment in vivo by increasing levels of PGC1a, which in turn is associated with the prevention of amyloid-β production [178], suggesting that SIRT1-mediated activation of PGC1a must be involved. HIV has been reported to decrease cellular NAD+ levels [179]. Zhang et al. [140] found reduction of NAD+ levels in cells exposed to Tat [140], which are related to SIRTs’ enzymatic activity. Furthermore, Tat-induced NAD+ depletion is mediated by miT-182 via down-regulation of NAMPT, which is the enzyme needed for the conversion of nicotinamide (NAM) to NMN [180]. 

## 7. Conclusions and Future Directions

Nowadays, PLWH can have a longer life expectancy thanks to cART. However, neuronal alterations are emerging that eventually will lead to HAND. Despite the fact that the milder forms are the most persistent in this population, it is necessary to find the molecular mechanisms governing neurodegeneration. Meanwhile, cART controls HIV pathogenesis, and there is evidence regarding its cytotoxicity effects. Additionally, infected cells that cross the BBB produce and secrete viral proteins that present neurotoxicity properties. Tat, a transactivator of HIV transcription, has a significant capacity to internalize into neurons causing neurotoxicity through several mechanisms such as ER stress and mitochondria dysfunction, which provoke cell death. Tat induces inhibition of SIRT1 and SIRT3 activities, meaning that Tat-mediated dysregulation of any of these molecules contributes to the neuropathogenesis of HAND; hence, these SIRTs are part of regulatory pathways that influence cell survival. Tat-induced neurotoxicity impairs these vias, so modulation of SIRTs is a promising therapy against neurodegeneration. Furthermore, studies have been conducted to test the available xenobiotics that regain the deacetylation activity of SIRTs in the context of neurodegenerative diseases and have obtained favorable results. It is known that SIRTs activators work by using different strategies; one the one hand, the exogenous modulators such as resveratrol activate SIRT1/SIRT3, while endogenous modulators such as NR increase NAD+ concentrations. In addition, it was reported that from the seven SIRTs, not only SIRT1 and SIRT3 could interact directly with Tat but also SIRT2. The effect of this interaction remains unclear even though SIRT2 also participates in the evolution of neurodegenerative diseases. In consequence, more studies are needed to elude inhibition/activation actions of all SIRTs in Tat-mediated neurotoxicity conditions so they can be taken collectively to generate the desired outcomes in the CNS after their modulation. Finally, despite cART being essential to maintain a low viral load, it cannot prevent the development of HAND, so adjuvant treatment is required. This additional therapy must be centered on regulators of hallmark altered pathways of neurodegeneration. Due to the current lack of specific medication for HAND and given that sirtuins are by no means pivotal enzymes in Tat-induced neurotoxicity, focusing on their modulation must be part of a therapeutic strategy to prevent and mitigate HAND.

## Figures and Tables

**Table 1:** Sirtuins activators.

Compound	Type	Modulation	Sirtuin	Effect	Stressor	Reference
Resveratrol	Natural polyphenol	Expression/ Activation	SIRT1 SIRT3	Increase: Mitophagy Mitochondria biogenesis Cell proliferation Decrease: Oxidative stress Apoptosis	LPS/GOx	[139]
					HIV-Tat	[140]
					Lead (Pb)	[141]
					Manganese (Mn)	[142]
					I/R	[106]
piceatannol	Natural Polyphenol /Resveratrol analog	Expression/ Activation	SIRT1	Increase: Mitochondrial biogenesis Decrease: Oxidative stress Apoptosis Inflammation	Antimycin A H_2_O_2_	[143]
					γ-irradiation	[144]
					I/R	[145]
Polydatin	Natural Polyphenol	Activation	SIRT3 SIRT1	Increase: Cell viability Antioxidant activity Autophagy Decrease: Oxidative stressApoptosis	Myocardial infarction	[146]
					Sulfur mustard	[147]
Honokiol	Natural polyphenol	Expression/ Activation	SIRT1 SIRT3	Increase: Mitochondria functions Decrease: Oxidative stress Apoptosis	Cardiac hypertrophy	[135]
					High glucose/fat diet	[148]
					I/R	[149]
Diosin	Natural saponin	Expression	SIRT1 SIRT3	Increase: Autophagy Decrease: Oxidative stress Apoptosis	Doxorubicin	[150]
					Oxyhemoglobin	[151]
					Aβ	[138]
Pyrroloquinoline quinone	Natural quinone	Expression/ Activation	SIRT1 SIRT3	Increase: Mitochondrial biogenesis NAD+	Wt	[152]
Melatonin	Hormone	Expression/ Activation	SIRT1 SIRT3	Increase: Antioxidant activity Mitochondria biogenesis Decrease: Oxidative stress Inflammation Apoptosis ER stress	ATH/Ox-LDL	[134]
					I/R	[133]
					Cadmium (Cd)	[153]
					LPS	[154]
					C/HI	[155]
					CoCl2	[156]
					Rotenone	[157]
Dihydromyricetin	Natural	Expression	SIRT3	Increase: Antioxidant activity Mitochondria dynamics Mitochondria biogenesis	TNF-α	[158]
β-lapachonequinone	Natural quinone	Expression	SIRT1	Increase: Activation of PGC1α Decrease: Oxidative stress	HD	[159]
Silybin	Natural flavonoid	Expression	SIRT3	Increase: Mitochondria function Decrease: Oxidative stress Apoptosis	Cisplatin	[160]
Metformin	Synthetic drug	Expression	SIRT3	Increase: Antioxidant activity Decrease: Oxidative stress	Type 2 diabetes	[161]
SRT2104	Synthetic drug	Expression/ Activation	SIRT1	Increase: Autophagy	TNF-α	[162]
SRT1720	Synthetic drug	Activation	SIRT1	Increase: Mitochondria biogenesis Antioxidant activity	High glucose	[163]
Tanshinone IIA	natural	Activation	SRT1	Increase: Energy production Mitochondria biogenesis Antioxidant activity NAD+ Decrease: Oxidative stress Mitochondria dysfunction Apoptosis Inflammation Ca^2+^ release HIV-1 transactivation	DNP	[164]
					HR	[165]
					Rotenone	[164]
					HIV-Tat	[166]

Abbreviations: (I/R): ischemic reperfusion injury; Aβ: amyloid-β peptide oligomer C/HI: cerebral hypoxia/ischemia; ATH: atherosclerotic model; HD: Huntington disease; HR: hypoxia/reoxygenation; TNF: tumor necrosis factor α; DNP: dinitrophenyl; ox-LDL: oxidized low-density lipoprotein; LPS: lipopolysaccharide; and GOx: glucose oxidase.

## Data Availability

Not applicable.
